# Supplementary material for: Cells grown in three-dimensional spheroids mirror in vivo metabolic response of epithelial cells
Source: Commun Biol. 2020 May 19;3:246. doi: 10.1038/s42003-020-0973-6 (PMC7237469; doi:10.1038/s42003-020-0973-6)
Supplement: Supplementary file 1 — Supplementary Information [file 42003_2020_973_MOESM1_ESM.pdf]

Supplementary Information

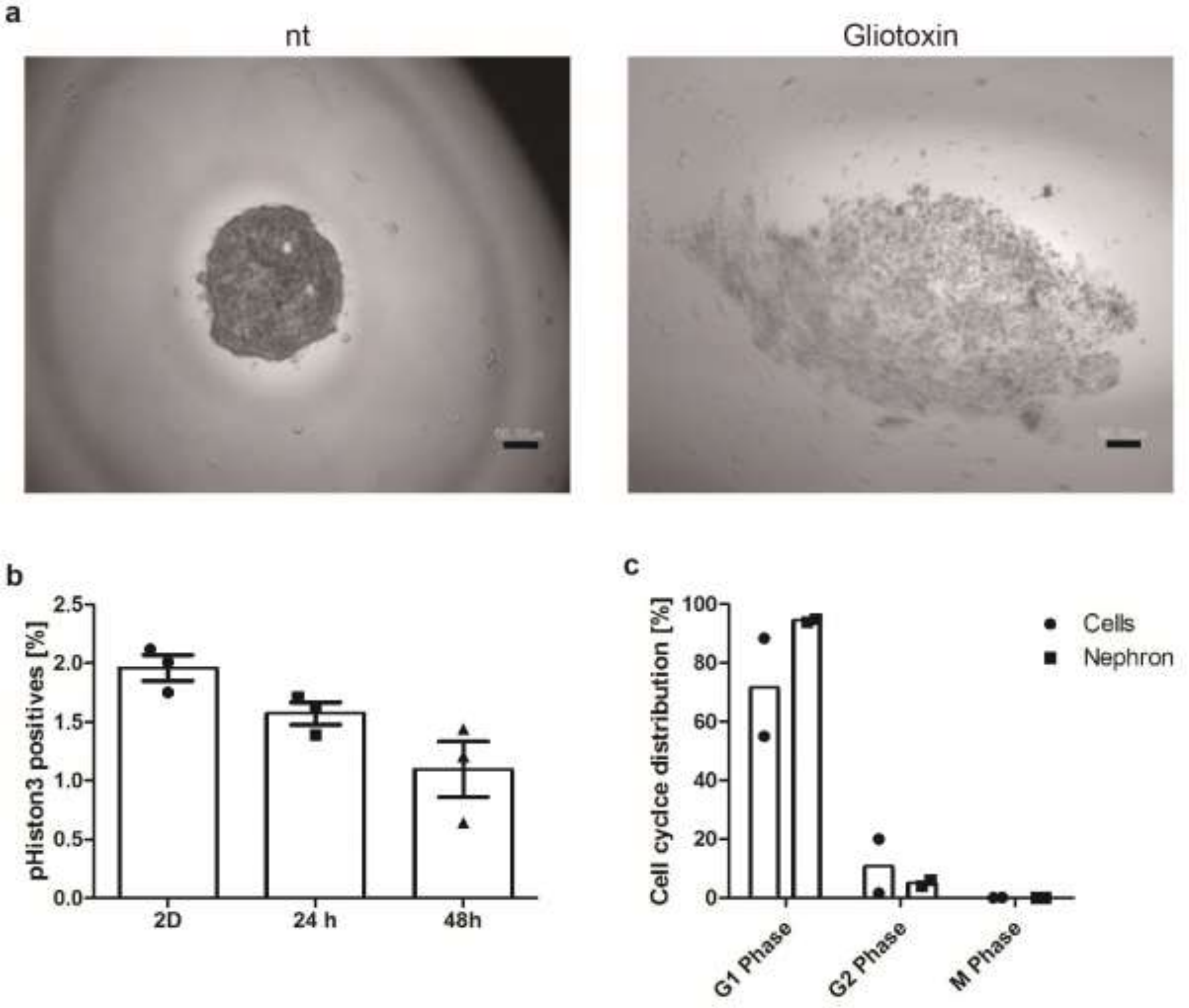

Supplementary Figure 1: In depth cell cycle analysis.

a, Cells only form spheroids when they express intact integrins on their surface. When integrin signalling is perturbed by the fungal secondary metabolite gliotoxin, cells cannot attach and die by anoikis. Size marker = 50 µm.

b, Phosphorylated Histone 3 serves as a marker for cells in mitosis. Spheroids show a decreased number of pHistone3 positive cells. N=3 individual flow cytometry analyses.

c, Comparison of cell cycle distribution of 3D grown spheroids and freshly isolated nephrons. Bars represent mean ± standard deviation of two independent FACS experiments.

Flow cytometry gating strategy for cell cycle analysis

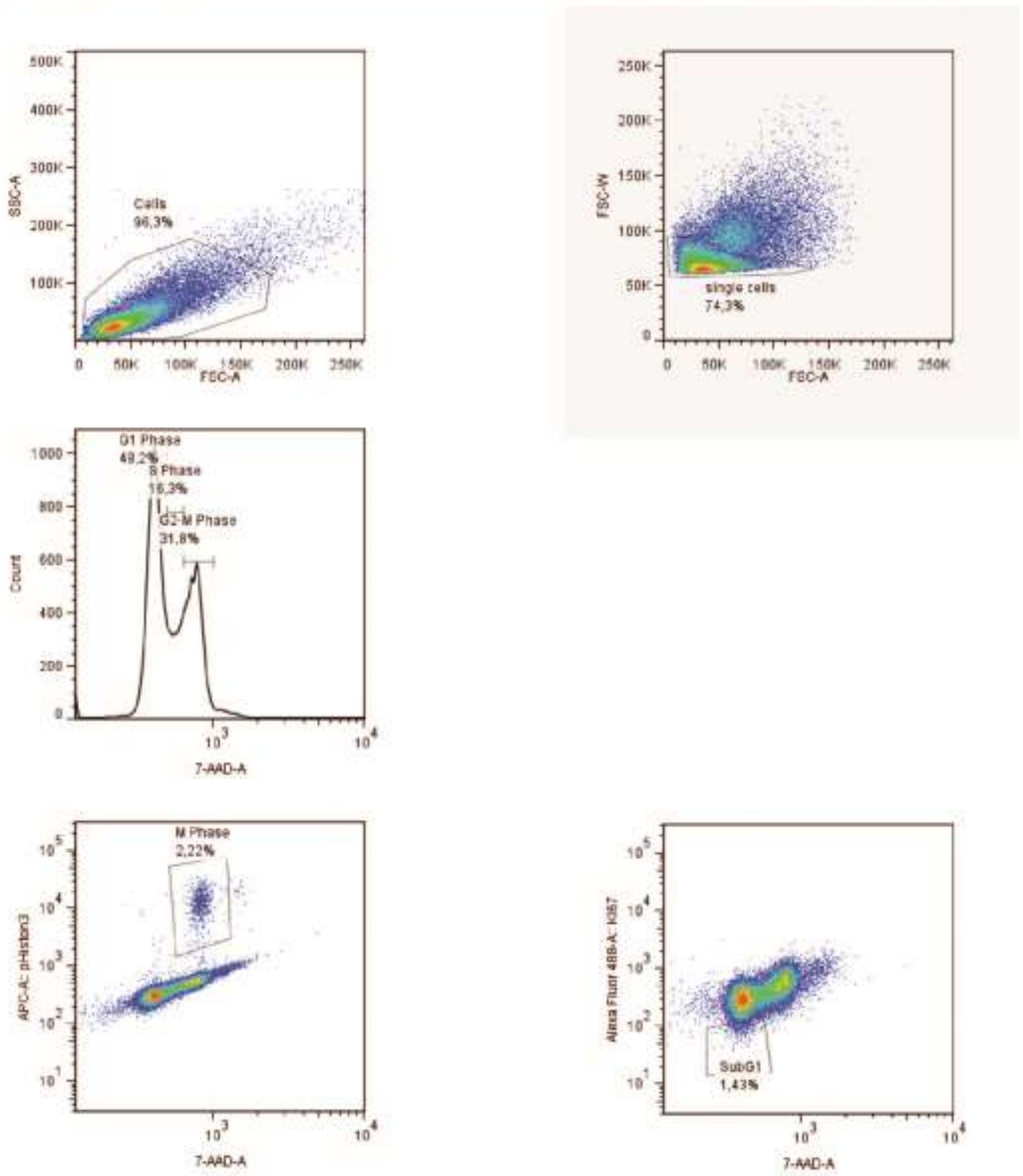

Supplementary Figure 2: Flow cytometry gating strategy for cell cycle analysis. Scatter blots and histograms showing the analysis strategy for the flow cytometry analysis of files recorded on Becton Dickinson LSRII and visualization on FlowJo, Version 7.6.5. After single cell selection, cells were gated on their DNA content using 7AAD as a marker allowing discrimination of G1, S and G2/M phase cells on a histogram. A co-staining for pHistone3-APC shows cells in M phase in the G2/M peak. A co-staining for Ki67-FITC reveals the proportion of G0 cells hiding in the G1 peak.

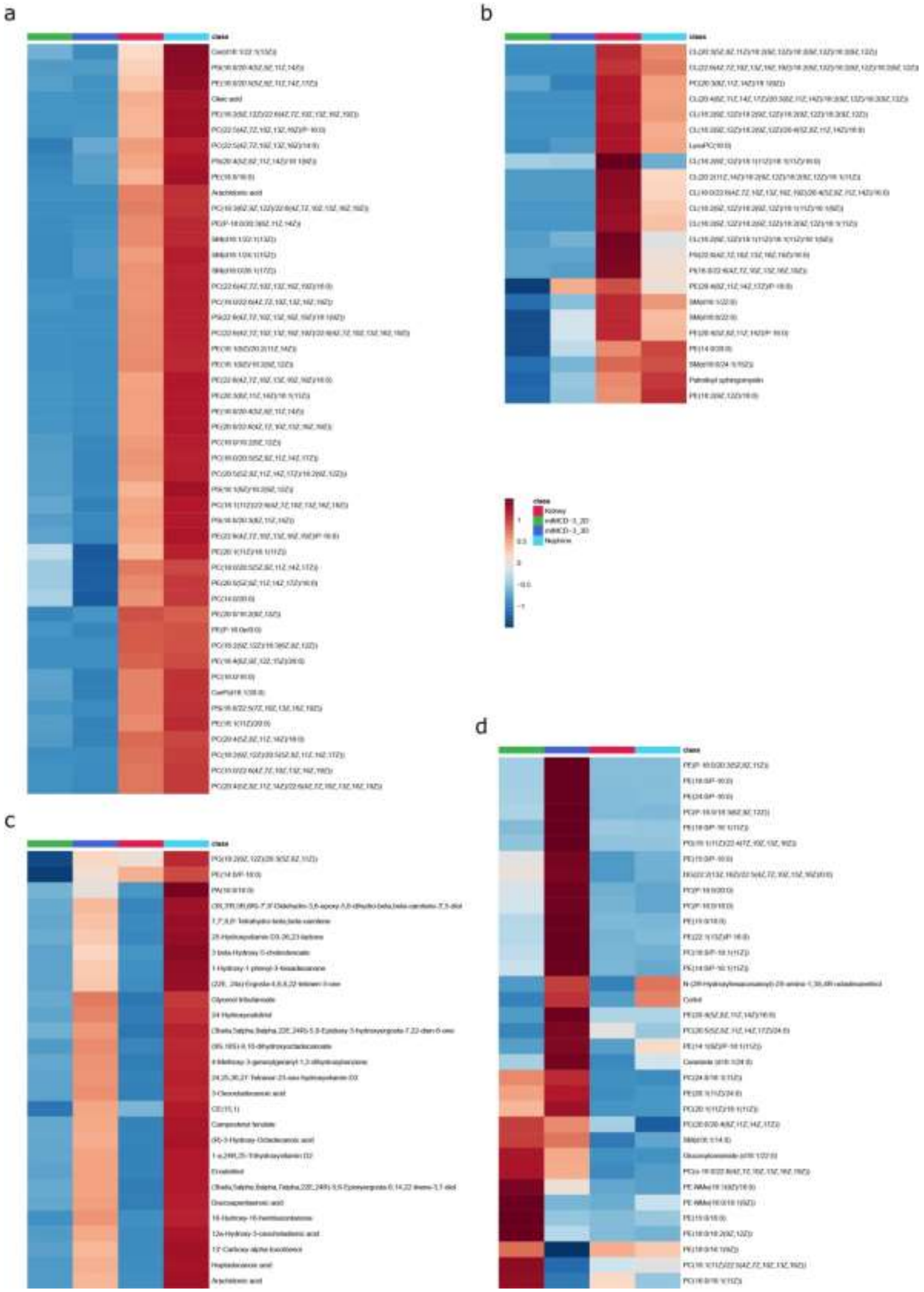

Supplementary Figure 3: Detailed heat map description from Figure 5b.

Range-scaled z-scores of annotated features with a q-value <0.05 according to ANOVA and FDR correction. For cluster analysis see figure 5.

a cluster a from Fig. 5.

b cluster b from Fig. 5

c cluster c from Fig. 5

d clusters d-f from Fig. 5

Green: 2D grown cells, red: whole kidney lysate, blue: 3D grown mIMCD3 cells, turquoise: isolated nephrons, n for nephrons =3, n for kidneys =4.

Original scans of the Western blots, Fig 4a

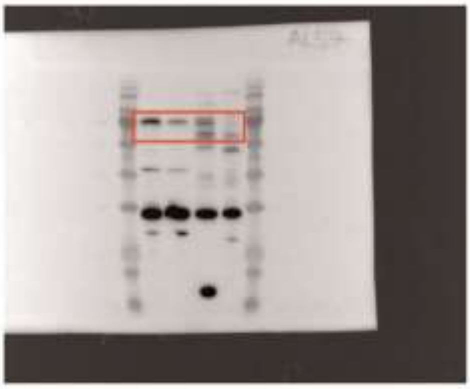

BGT2

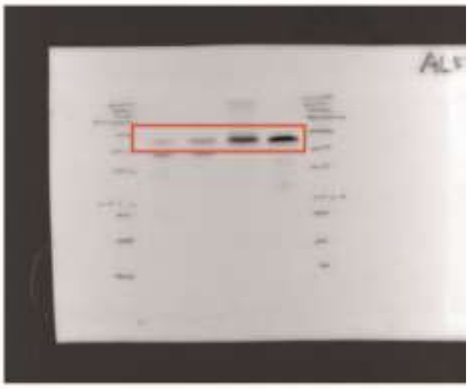

Tubulin

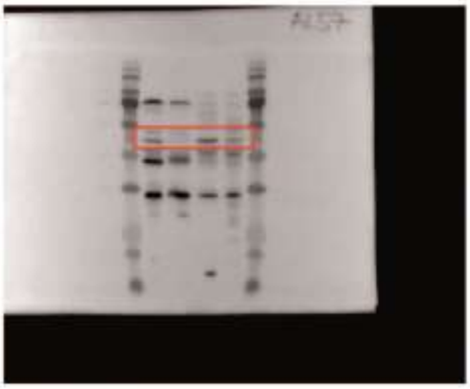

PCYT

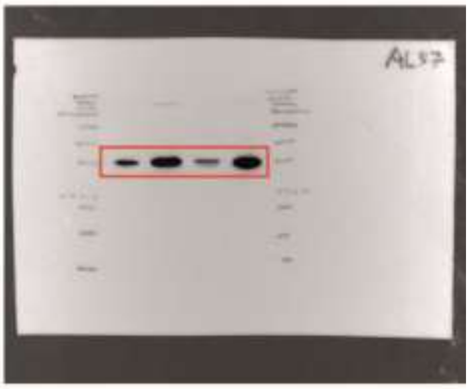

GAPDH

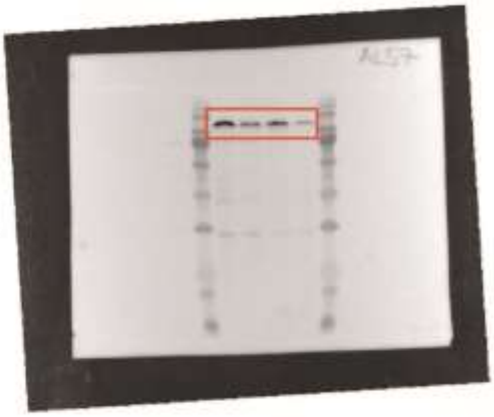

Hexokinase 1

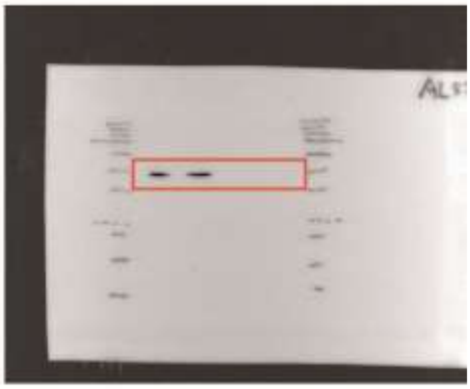

Actin

Supplementary Figure 4: Uncropped images of Western blots for Fig. 4a
