# Supplementary material for: Cells grown in three-dimensional spheroids mirror in vivo metabolic response of epithelial cells
Source: Commun Biol. 2020 May 19;3:246. doi: 10.1038/s42003-020-0973-6 (PMC7237469; doi:10.1038/s42003-020-0973-6)
Supplement: Supplementary file 2 — Description of Additional Supplementary Files [file 42003_2020_973_MOESM2_ESM.pdf]

Description of Additional Supplementary Items

File name: Supplementary Table 1
Description: Sequences of qPCR primers

File name: Supplementary Data 1
Description: All the source data of blot figures presented in the main and supplementary figures

File name: Supplementary Data 2
Description: Measured metabolites from 2D/3D cell lysates

File name: Supplementary Data 3
Description: Measured excreted metabolites from 2D/3D cell lysates

File name: Supplementary Data 4
Description: Measured metabolites from 2D/3D/kidney/nephron lysates

File name: Supplementary Data 5
Description: Measured lipids from 2D/3D/kidney/nephron lysates

File name: Supplementary Data 6
Description: Statistics on metabolites from 2D/3D cell lysates

File name: Supplementary Data 7
Description: Statistics on excreted metabolites from 2D/3D cell lysates

File name: Supplementary Data 8
Description: Statistics on metabolites from 2D/3D/kidney/nephron lysates

File name: Supplementary Data 9
Description: Statistics on lipids from 2D/3D/kidney/nephron lysates
