# Supplementary material for: Cells grown in three-dimensional spheroids mirror in vivo metabolic response of epithelial cells
Source: Commun Biol. 2020 May 19;3:246. doi: 10.1038/s42003-020-0973-6 (PMC7237469; doi:10.1038/s42003-020-0973-6)
Supplement: Supplementary file 13 — Reporting Summary [file 42003_2020_973_MOESM13_ESM.pdf]

nature research

Corresponding author(s): Lukas Peintner

Last updated by author(s): Apr 15, 2020

Reporting Summary

Nature Research wishes to improve the reproducibility of the work that we publish. This form provides structure for consistency and transparency in reporting. For further information on Nature Research policies, seeAuthors & Referees and theEditorial Policy Checklist .

Statistics

For all statistical analyses, confirm that the following items are present in the figure legend, table legend, main text, or Methods section.

n/a	Confirmed	
☐	☒	The exact sample size (*n*) for each experimental group/condition, given as a discrete number and unit of measurement
☐	☒	A statement on whether measurements were taken from distinct samples or whether the same sample was measured repeatedly
☐	☒	The statistical test(s) used AND whether they are one- or two-sided<br>*Only common tests should be described solely by name; describe more complex techniques in the Methods section.*
☒	☐	A description of all covariates tested
☐	☒	A description of any assumptions or corrections, such as tests of normality and adjustment for multiple comparisons
☐	☒	A full description of the statistical parameters including central tendency (e.g. means) or other basic estimates (e.g. regression coefficient) AND variation (e.g. standard deviation) or associated estimates of uncertainty (e.g. confidence intervals)
☒	☐	For null hypothesis testing, the test statistic (e.g. *F*, *t*, *r*) with confidence intervals, effect sizes, degrees of freedom and *P* value noted<br>*Give P values as exact values whenever suitable.*
☒	☐	For Bayesian analysis, information on the choice of priors and Markov chain Monte Carlo settings
☒	☐	For hierarchical and complex designs, identification of the appropriate level for tests and full reporting of outcomes
☒	☐	Estimates of effect sizes (e.g. Cohen's *d*, Pearson's *r*), indicating how they were calculated

Our web collection on statistics for biologists contains articles on many of the points above.

Software and code

Policy information about availability of computer code

Data collection	Flow cytometry: Beckton Dickinson Diva<br>Microscopy: ZEISS ZEN blue software<br>qPCR: Bio Rad CFX Manager
Data analysis	Analysis of MS data: ProgenesisQI<br>Analysis of MS data: MetaboAnalyst 4.0<br>Analysis of qPCR Data: Bio Rad software<br>Analysis of FACS Data: FlowJo

For manuscripts utilizing custom algorithms or software that are central to the research but not yet described in published literature, software must be made available to editors/reviewers. We strongly encourage code deposition in a community repository (e.g. GitHub). See the Nature Research guidelines for submitting code & software for further information.

Data

Policy information about availability of data

All manuscripts must include a data availability statement. This statement should provide the following information, where applicable:

- Accession codes, unique identifiers, or web links for publicly available datasets
- A list of figures that have associated raw data
- A description of any restrictions on data availability

All data generated or analysed during this study are included in this published article (and its supplementary information files).

Field-specific reporting

Please select the one below that is the best fit for your research. If you are not sure, read the appropriate sections before making your selection.

- [x] Life sciences
- [ ] Behavioural & social sciences
- [ ] Ecological, evolutionary & environmental sciences

For a reference copy of the document with all sections, see nature.com/documents/nr-reporting-summary-flat.pdf

Life sciences study design

All studies must disclose on these points even when the disclosure is negative.

Sample size	Sample size was set to n=4 due to the best practice in the laboratory where the measurements where conducted.
Data exclusions	No data has been excluded.
Replication	qPCR measurements and sample preparation has been repeated in 3 independent experiments.
Randomization	Randomization was not relevant to this study
Blinding	The first author was entireley blinded on all the samples that were measured. The blinding of the samples was conducted by the senior author.

Reporting for specific materials, systems and methods

We require information from authors about some types of materials, experimental systems and methods used in many studies. Here, indicate whether each material, system or method listed is relevant to your study. If you are not sure if a list item applies to your research, read the appropriate section before selecting a response.

Materials & experimental systems

n/a	Involved in the study	
[ ]	[x]	Antibodies
[ ]	[x]	Eukaryotic cell lines
[x]	[ ]	Palaeontology
[ ]	[x]	Animals and other organisms
[x]	[ ]	Human research participants
[x]	[ ]	Clinical data

Methods

n/a	Involved in the study	
[x]	[ ]	ChIP-seq
[ ]	[x]	Flow cytometry
[x]	[ ]	MRI-based neuroimaging

Antibodies

Antibodies used

Ki67: Santa Cruz sc7846, lot#L2104
p-Histone3: BD Alexa Fluor 647 rat anti Histone H3C (pS28), Clone: HTA28, Cat#558217, Lot#7172813
Hexokinase 2: Cell signaling (C64G5), Cat#2867S Lot#5
Bgt1: ab200676, Lot#GR3203423-2
PCYT2: ab15053, Lot#GR308764-2
Actin: MP Biochemicals, #69100, Dilution 1:20.000
Tubulin: Bio Rad, MCA77G
GAPDH: Cell signaling #5174

Validation

Ki67 and p-Histone3: Peintner et al 2015
Hexokinase 2: Kshattry et al 2019
Bgt1: Ren et al 2019
PCYT2: No published validation so far
Actin, Tubulin and GAPDH: Haun et al 2018

Eukaryotic cell lines

Policy information about cell lines

Cell line source(s)	All cell lines were initially purchased from ATCC CRL-2123

Authentication	Leibnitz Institute, Deutsche Sammlung von Mikroorganismen und Zellkulturen GmbH, Germany
Mycoplasma contamination	The mIMCD3 cells were tested negative for mycoplasma contamination (performed by PCR in our laboratory)
Commonly misidentified lines (See ICLAC register)	n/a

Animals and other organisms

Policy information about studies involving animals; ARRIVE guidelines recommended for reporting animal research

Laboratory animals	Mus musculus, C57BL/6N, females, 6-8 weeks old
Wild animals	Study did not involve wild animals
Field-collected samples	Study did not involve Field-collected samples
Ethics oversight	No ethical approval was necessary for the study; The permission to extract organs without previous treatment of the animal was granted by the Regierungspräsidium Freiburg to the Laboratory of the senior author.

Note that full information on the approval of the study protocol must also be provided in the manuscript.

Flow Cytometry

Plots

Confirm that:

- [x] The axis labels state the marker and fluorochrome used (e.g. CD4-FITC).
- [x] The axis scales are clearly visible. Include numbers along axes only for bottom left plot of group (a 'group' is an analysis of identical markers).
- [x] All plots are contour plots with outliers or pseudocolor plots.
- [x] A numerical value for number of cells or percentage (with statistics) is provided.

Methodology

Sample preparation	Detailled description of the sample preparation is listed in the method section of the manuscript.
Instrument	Beckton Dickinson LSRII
Software	Beckton Dickinson DIVA for recording;<br>FlowJo for analysis
Cell population abundance	n/a
Gating strategy	Selecting living cells in FSC/SSC, gating on single cells in FSC-A/FCS-W, then histogram on 7AAD to discriminate between G1, S and G2/M phase, blot 7AAD against pHiston3-APC to visualize M-Phase cells, blot 7AAD against Ki67-FITC to measure Sub-G1 cells.

- [x] Tick this box to confirm that a figure exemplifying the gating strategy is provided in the Supplementary Information.
